# Deep learning–based region merging with adaptive threshold optimization for building segmentation in remote sensing images

**DOI:** 10.1371/journal.pone.0348364

**Published:** 2026-05-15

**Authors:** Asim Shoaib, Muhammad Waqas Nadeem, Nohaidda Sariff, Syeda Tehreem Haider, Fasee Ullah, Ateeq Ur Rehman, Sadiq Muhammad, Rab Nawaz, Muhammad Adnan Khan

**Affiliations:** 1 Faculty of Information and Communication Technology, Universiti Tunku Abdul Rahman, Kampar, Perak, Malaysia; 2 School of Engineering, Faculty of Innovation and Technology, Taylor’s University, Malaysia; 3 Department of Electronics Engineering, Faculty of Electronics and Electrical Engineering, University of Engineering and Technology, Taxila, Pakistan; 4 Department of Computing, Universiti Teknologi Petronas, Seri Iskandar, Perak, Malaysia; 5 Computer Science and Engineering, Saveetha School of Engineering, Saveetha Institute of Medical and Technical Sciences, Chennai, Tamilnadu, India; 6 Applied Science Research Center, Applied Science Private University, Amman, Jordan; 7 School of Computing, Gachon University, Seongnam-si, Republic of Korea; 8 School of Computer Science and Electronic Engineering, University of Essex, Colchester, Essex, United Kingdom; 9 Riphah School of Computing and Innovation, Faculty of Computing, Riphah International University, Lahore Campus, Lahore, Pakistan; 10 Jadara University Research Center, Jadara University, Irbid, Jordan; 11 Center of Excellence in Precision Medicine and Digital Health, Department of Physicology, Faculty of Dentistry, Chulalongkorn University, Bangkok, Thailand; Universidade Federal de Uberlandia, BRAZIL

## Abstract

Precise extraction of buildings from high-resolution remote sensing images is essential for urban analysis and land management. However, accurately extracting buildings as a region of interest (ROI) from remote sensing (RS) images remains challenging. This difficulty arises from the spectral similarity of other objects, such as roads, cars, or trees, along with limited information on building boundaries and small buildings. Traditional image segmentation methods often rely on a fixed threshold value, making optimisation difficult in cases of over-segmented regions. As a result, region merging is subsequently performed on the region adjacency graph (RAG). Consequently, building segmentation in RS images becomes problematic and can lead to inaccurate boundary delineation or region classification. To overcome these limitations, we propose a novel segmentation approach that incorporates an adaptive thresholding optimisation technique and a merging criterion (MC) based on deep features extracted via a convolutional neural network (CNN)-based AttentionU-Net architecture. This ensures that merging decisions are guided by intrinsic region-level characteristics and refined through deep feature representations. Beginning with initial segmentation generated by the simple linear iterative clustering (SLIC) algorithm, the AttentionU-Net architecture is applied to high-resolution RS images to extract deep features, respectively. As a result, our approach combines both low and high-level feature information, reducing misalignment during merging and enhancing traditional region merging strategies. To validate this approach, the WHU buildings’ RS images dataset was utilised. Experimental results demonstrate that our approach achieves superior segmentation accuracy in building delineation while eliminating the need for rigid thresholds. Finally, the results were compared with those obtained using the multiresolution segmentation (MRS) algorithm implemented in eCognition software on the same WHU buildings RS images, where our approach performs better. Specifically, the proposed approach attained a higher segmentation accuracy, with an F-measure of 0. 91 and a goodness of segmentation score G_s_ of 0.92, compared to 0.52 and 0.83, respectively, achieved by the MRS algorithm.

## 1. Introduction

Advancements in satellite remote sensing (RS) technology have made it easier to acquire high-resolution images across large regions. Accurate extraction of buildings is crucial for land mapping and urban planning. Building extraction techniques from high-resolution RS images can be divided into two groups: conventional and deep learning-based image segmentation approaches. Deep learning-based segmentation methods classify pixels in images into geo-objects perfectly [1]. However, they only segment specific classes such as buildings, roads, or vegetation, and do not provide a global context of other geo-objects as compared to conventional image segmentation algorithms. Moreover, conventional image segmentation approaches primarily use geometric, spectral, and textural features [2], such as edge-based [3] and region-based segmentation algorithms [4]. Moreover, these algorithms may perform well in handling simple scenes. However, they may find it difficult to meet the accuracy requirements of building extraction tasks due to the continuous complexity and high resolution in RS images.

Conventional image segmentation algorithms are effective in the process of dividing images into regions with similar shapes and sizes [5]. However, when it comes to the entire geo-object of RS images, conventional image segmentation algorithms are less effective. Additionally, these conventional image segmentation algorithms may need to be manually and expertly interpreted using the building’s ROI statistical characteristics in RS images [6]. Hence, it might be challenging to apply to larger RS image datasets. Moreover, these region-based segmentation algorithms lead to over-segmentation (OS) [7]. OS means the creation of small regions that do not provide meaningful segmented object regions in the image [8]. Hence, to prevent the OS in the region-based segmentation algorithms, the region merging approach is commonly used [9].

Region merging approaches have been proposed in literature [10–13] that are aimed at completing geo-object segmentation in RS images. Region merging is commonly performed by iteratively merging the over-segmented regions to extract the ROI [10]. Since this approach operates at the region level, it captures detailed information about object features for merging, compared to methods that work at the pixel level [13]. Furthermore, in the region merging approach, the merging is conducted based on the similarity or dissimilarity of the features of regions selected for merging [14]. This measure of regions’ features similarity, or dissimilarity, is known as the MC [15]. Region merging can be performed using a graph known as the region adjacency graph (RAG) [16]. In RAG, the over-segmented regions are the nodes, while the MC depicts the edge connecting these nodes. Pairs of regions are merged iteratively until the MC and optimization are satisfied [17].

Moreover, region merging approaches enhance the overall accuracy of segmentation and avoid compromising the quality of the OS. Additionally, the quality of the OS used for similar computation, i.e., MC, and the proper choice of the scale parameter, which is the threshold, have a significant impact on the segmentation accuracy of region merging approaches [1]. When utilizing improper scale parameters, which are the threshold values, there is a possibility of resulting in an OS error. To address these issues, there is a need for more complex strategies to improve the threshold optimization and the MC technique to increase the building ROI extraction or delineation accuracy.

The key contributions of our study are as follows.

1) This study proposes a novel approach for enhancing the optimization process in the region merging approach. This optimization process is derived from the deep features extracted from a CNN-based deep learning (DL) architecture. It oversees the overall as well as at each single merging step without being limited to any hard threshold value.
2) The MC is derived from a combination of three and four different features to find the optimal final segmentations.
3) Additionally, many experiments have been performed to analyze the OS, features for MC, and optimization for the most suitable combination of segmentation region merging approach.

The organization of this paper is given as follows. Section 2 provides the relevant studies, Section 3 presents the experimental results, Section 4 presents the discussions, and Section 5 concludes the paper.

## 2. Literature review

Image segmentation plays an essential role in RS image analysis workflows. Many region-based segmentation algorithms exist; including the Felzenszwalb and Huttenlocher (FH) [18], Mean Shift (MS) algorithm [19], Quick Shift (QS) [20], simple linear iterative clustering (SLIC) algorithm [21], region growing [22], watershed transform [23], Compact Watershed (CW) [24], and random walk [25]. Among the algorithms mentioned, SLIC [21] effectively delineates the regions in the image compared to other region-based segmentation algorithms [26]. This is because in SLIC, a parameter known as the number of centroids, k, must be defined by the user. It controls the number of regions that the algorithm will segment the image into, allowing for improved segmentation accuracy [21]. A higher value of parameter k value 3000 [27] results in smaller segments of regions being generated, while a lower k value of 250 produces larger numbers of regions [28]. Generally, these region-based segmentation algorithms begin by identifying pixels having similarity of feature values, then traverse until they reach the edge pixels of object regions for delineation, but suffer from severe OS [29]. To solve OS problems, region merging approaches have been commonly proposed in the literature.

In a study [10], there is an introduction of a hybrid region merging (HRM) technique that shows integration of both the global and local oriented region merging strategies used for high-resolution RS image segmentation. The approach used in [10] starts by selecting the globally most similar pair of regions to start the merging process, enhancing the optimization as compared to the local-features extraction approaches. Subsequent merging iterations are then constrained to the local neighborhood, which accelerates segmentation and better captures local context. This approach leverages a graph model that is built from the initial OS and uses features such as region size, compactness, homogeneity, and edge strength for the guidance of merging decisions [10]. Another study [30] has employed a region merging technique for high-resolution RS image segmentation, focusing on the impact of dynamically shifting seed regions during the merging process. They compare different merging strategies, such as local best merging (LBM), local mutual-best merging (LMM), and global best merging (GBM), and demonstrate that allowing seed regions to shift dynamically leads to more even region expansion and improved segmentation accuracy. Their method uses a graph-based model built on an initial OS, with similarity measures incorporating region size, spectral, shape, and edge features to guide the merging decisions [30]. For region merging, another study [31] has proposed a hybrid segmentation method that uses local spectral-angle thresholds. To improve accuracy and the multi-scale segmentation, it adapts to local spectral variation. Although it achieved more realistic object boundaries than the global methods. However, it still requires threshold tuning and more computation. For high-resolution RS images. Another research [32] proposed an adaptive scale-variable region merging algorithm that can estimate local scale parameters for segmenting objects of different sizes. Algorithms are tested on three GF-2 satellite scenes. The approach achieved lower total error (TE) and higher overall segmentation quality (OSQ) scores than other methods in the previous literature, thus producing multi-scale segmentation with more accuracy. Previous research study in [33], proposed a local scale-guided hierarchical method with OS and under segmentation (US) correction for region merging. By using normalized difference vegetation index (NDVI)-based analysis, the model achieved lower TE and quality rate (QR) values on Gaofen-1 images. Moreover, their approach shows a higher segmentation accuracy result. An optimized region merging algorithm [34] combining tiling, adaptive Gaussian filtering, and stability margin concepts for efficient segmentation. This approach achieved an improved accuracy, and it provided 20–30% faster processing as compared to conventional approaches. In [35], authors proposed strategy global merging costs and stepwise optimization for the hierarchical region-merging algorithm to segment individual trees from unmanned aerial vehicle (UAV) and LiDAR point clouds. Across the forests of varying densities, this model has achieved high F-scores of approximately 0.80 to 0.91. It has outperformed traditional watershed and region-growing methods, especially in the dense and mixed forests datasets. This type of region merging approach shows excellent results. However, it may not be possible to apply very high-resolution and a large number of RS images very quickly. Thus, the researchers employed machine learning and DL to propose different strategies in region merging approaches. Comparative analysis of previous region merging approaches has been demonstrated in Table 1.

**Table 1:** Comparative analysis of previous region merging (RM) approaches.

Reference	Brief Summary	Key Methods	Pros	Cons
[1]	DL-based RM framework	DL and RAGs integration with weakly supervised multiscale feature learning.	Robust to shape/scale Acc. (F = 0.9552, TE = 0.0827);	Computationally intensive, requires deep model tuning
[15]	Unsupervised deep segmentation using dense features + RM	Combines dense FCN features with shallow features	Unsupervised, label-free, achieves accurate segmentation.	Tested only on BSD300, computationally heavy
[31]	Hybrid segmentation with spectral-angle thresholds for RM	Multi-band watershed, RAG, spectral-angle distance, and heterogeneity-based RM	Higher acc., adaptive to local variation, multi-scale results	Needs threshold tuning, more computation, not fully automatic.
[32]	Scale-adaptive RM for high-res remote-sensing images.	Coarse half-size segmentation, local SP estimation, and adaptive merging	Accurately segments multi-scale objects, uses contextual info via super-segments	Struggles with small objects, sensitive to parameters (ρ and SP global)
[33]	Scale-guided RM with segmentation correction.	Watershed + NDVI + scale and CV-based correction	Accurate & adaptive, minimizes errors, efficient	Hyperparameter tuning needed,
[34]	Optimized RM for large images.	Tiling + adaptive filtering + RAG-NNG merging.	High acc., 20 - 30% faster, noise-robust, scalable.	Limited data, unclear, no DL
[35]	Hierarchical RM for individual tree segmentation.	Density-based OS; global compactness RM	High accuracy 80-91%, detects 10% more trees	Parameter-dependent, limited to forest datasets.
**Our Approach**	Proposing deep learning-based threshold and MC optimization	Deep features utilized for optimization such as threshold and MC	Precise buildings ROI segmentation	Requires the SLIC k parameter value initially to generate the OS

Previous study [11] introduced a region merging approach for RS images that uses a machine learning strategy, which utilizes a random forest (RF) classifier to guide merging decisions instead of using a single value for threshold. By incorporating different combinations of features and deriving multiple MCs, their approach improves accuracy for building ROI segmentation. The approach also includes an efficient sample collection strategy for training the RF classifier, demonstrating strong performance across various high-resolution satellite datasets [11]. In another study [1], a deep learning-based region merging approach has been proposed for building segmentation in RS images. It starts with an initial OS, then employs a transformer-based network (S2Former) to learn the similarity between adjacent OS regions. The MC is derived from features extracted by DL, and merging of regions is performed with the threshold value set at 0.5. This approach enables accurate, weakly supervised segmentation of geo-objects of varying sizes in very high-resolution RS images, showing promising results as compared to machine learning or conventional region merging approaches [1]. The study [15] proposed a bottom-up, unsupervised image segmentation approach that combines DL features representation with a statistical region merging (SRM) algorithm. Their approach first uses a fully convolutional dense U-Net (FCD U-Net) to extract deep features and generate an over-segmented image. The training of the FCD U-Net is iteratively performed until either a maximum of 10 iterations is reached or the improvement in loss falls below a threshold of 0.07, ensuring efficient convergence. In the second phase, a high-dimensional feature-based region merging strategy, guided by a region adjacency graph (RAG), is applied to iteratively merge similar regions and produce the final segmentation. This hybrid approach leverages both deep feature learning and traditional region merging for accurate and spatially coherent results [15]. However, the conventional thresholding strategies have often failed to generalize across diverse scenes in RS images, resulting in incomplete building segments.

Additionally, some of the previous research works referenced as [36,37] have improved the semantic segmentation by proposing improved DL architectures. These works extract the features in the encoder part and utilized them in either multi-scale segmentation or in contextual segmentation strategies to perform ROI delineation. Even though these deep learning-based methodologies have offered promising results, the approaches are more towards improving DL segmentation approaches. Whereas there is a limited concept of improvement in OS issues through traditional region merging approaches by utilizing DL, as in most of the previous studies, it is vice versa. It is true that DL still requires large, annotated datasets, and it only focuses on specific ROI in RS image segmentation. Moreover, those traditional region merging approaches that have not utilized DL strategies are missing local region characteristics that are essential for thresholding optimization and MC derivation. Furthermore, most of the existing region merging approaches either rely entirely on pixel-level information or apply global or hard value thresholds, which are difficult when dealing with spatial and contextual variations within RS imagery. Based on this, there is room for proposing a region merging approach that can solve OS, capture fine-grained region boundaries, and intelligently merge regions based on meaningful MC and threshold optimization by utilizing DL extracted features.

## 3. Materials and methods

### 3.1. Dataset description

In this study, a publicly benchmarked RS images dataset known as the WHU buildings dataset [38] was chosen. The dataset was released in 2019 and is publicly available on the link [12]. It consists of 0.075-m resolution aerial images of Christchurch, New Zealand, covering a 450 km^2^ area. It contains a total of 8189 images, each with dimensions of 512 × 512 pixels. The dataset has original images with the corresponding ground truths (GTs). This dataset was selected as it provides detailed spatial and geographical information of visual object regions in the images [39]. Moreover, this dataset contains a collection of remote sensing images from multiple sources, including satellite and aerial images [38], since the focus of this study is to delineate building region as ROI from the dataset. The images and their corresponding GTs that either display limited building regions or contain no building regions have been excluded from this research work.

### 3.2. Proposed approach

**Fig 1:**
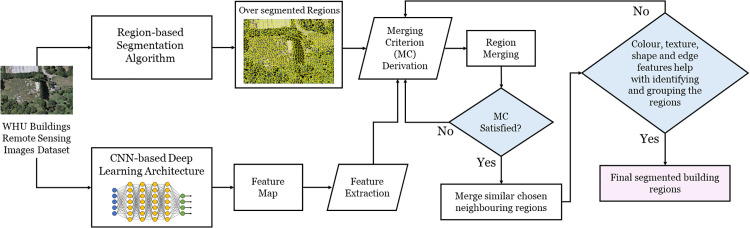
The proposed framework for the region merging approach.

Fig 1 represents the flowchart for the region merging optimization through a deep learning-based extracted features threshold and derivation of MC, respectively. The proposed approach is designed on an experimental flow which involves generating the over-segmented regions, followed by extracting the feature map from the AttentionU-Net CNN-based DL architecture. These extracted features are then used to propose an adaptive threshold and MC for merging building ROIs in RS images. This process aims to address OS and improve building delineation, while eliminating the requirement of human intervention in the merging process. The experiments are conducted using Python v3.10.4. Hardware specifications are mentioned in Table 2.

**Table 2:** Hardware Specifications.

Details	Operating System	IDE	RAM	GPU
Personal Computer	Windows 10	Google Colab Pro+ (Purchased version)	12 GB	84 GB
Laptop (Intel Core i5)	Windows 10	Spyder	8 GB	3.9 GB shared memory

The first step in the implementation of the proposed region merging approach involves the implementation of a region-based segmentation algorithm on the WHU Buildings RS images dataset [38]. In our previous research study [40], we compared the four region-based segmentation algorithms, which are Felzenszwalb and Huttenlocher (FH), Compact Watershed (CW), Quick Shift (QS), and SLIC, on the WHU Buildings RS images dataset. Their experimental results indicate that SLIC performance is better for generating initial OS on WHU Buildings RS images. The purpose of the comparison was to select the best-performing region-based segmentation algorithm in generating the over-segmented regions, which are well-aligned with the image object regions’ boundaries in the WHU Buildings RS images. Based on this comparison, we select the SLIC algorithm for generating an initial OS for our proposed region merging approach.

Secondly, a CNN-based DL architecture has been selected in this study for generating the feature map of buildings as ROIs based on the results of experiments among five encoder-decoder DL architectures in our previous research study referenced in [41]. We used the WHU Buildings dataset for comparison among five encoder-decoder DL architectures, which have been utilized in this research study. The results of that study show that AttentionU-Net is better than U-Net [38], SegNet [42], V-Net [43], and ResU-Net [44]. Thus, the AttentionU-Net has been employed on the WHU Buildings RS images dataset to delineate buildings as ROI for the implementation of the proposed region merging approach in this study.

In our experimentation third step, the four features that are colour, texture, shape, and edge, which are extracted from the ROI of buildings resulting from the DL architecture, are treated as a dimensional feature embedding vector. These features were then used to optimize the merging process on the over-segmented regions. After buildings an ROI delineation through DL architecture, the four features are stacked through horizontal stacking in the forthcoming step [45]. According to the authors in [45], horizontal stacking is the process where different features having different dimensions are stacked as one after another. In this way, the colour $C_{I_{r}\;\in \;I}$, texture $G_{I_{r}\;\in \;I\;}$, shape ${\;A}_{I_{r}\;\in \;I}$, and edge features ${ED}_{I_{r}\;\epsilon \;I}$ are horizontally stacked, each after one another. In the subsequent RAG graph construction, each of the six feature channels in $F_{I_{r}\in I}$ is first linearly scaled to the range [0, 1] within the image so that colour texture, shape, and edge information are comparable in magnitude. Moreover, each scalar feature channel is linearly normalized and within the image range, as stated above, to avoid scale dominance of any single cue. The single scalar dissimilarity between two neighboring regions $I_{r}$ and $I_{j}$ is then computed as the Euclidean distance. Furthermore, the merging process will use these stacked features when the difference in these feature values (colour, texture, shape, and edge information) between neighboring regions is considered for region merging. Thus, the stacked vector provides a compact and comparable descriptor that allows the four features to be adjusted into a single scalar dissimilarity for each pair of adjacent regions through Euclidean distance. The features vector can be represented as in equation 1.


$$\begin{array}{c} {\;F}_{I_{r}\;\in \;I}=hstack(C_{I_{r}\;\in \;I}\;,G_{I_{r}\;\in \;I\;},{\;A}_{I_{r}\;\in \;I},{\;ED}_{I_{r}\;\in \;I}) \end{array}$$ (1)

Where $F_{I_{r}\;\in \;I}$ are the features from over-segmented regions, and hstack refers to the horizontal stacking of four features as stated above. The dimension of $F_{I_{r}\;\in \;I}$ will be 1 x 6, which has horizontal stacking where 6 rows and 1 column. The 3 dimensions are for colour, having mean of R, G, B channels, 1 for texture feature resultant from Gabor matrix as mean, 1 for shape as area attribute having number of pixels, and 1 for edge information as indices.

The next step involves defining the threshold in order to set a limit on merging edges and employing it in MC. According to a previous study [46], the authors stated that due to the high variability of the RS image features, utilization of a static value or hard threshold affects the overall segmentation efficiency [15]. For each region $I_{l}$, the stacked feature vector $F_{I_{l}\in I}$ is computed as in (1) but restricted to the pixels inside the AttentionU-Net generated building as ROI. The $\mu (F_{I_{l}\in I})$ in (2) denotes the arithmetic mean of these region-level feature magnitudes over all ∣K∣ building regions. Moreover, multiplying by ∣K∣makes the threshold proportional to the total features, also stacked in eq (1) associated with buildings in the current ROI. In this way, th is not a fixed, hand-tuned constant value but each building as an ROI and scene-adaptive quantity that reflects the statistics of the AttentionU-Net based building ROIs. Wherever AttentionU-Net predicts more numerous or more heterogeneous buildings as ROIs, it yields a higher threshold (allowing more aggressive merging to recover complete extents). Whereas building ROIs with fewer and more homogeneous results in a lower threshold value and thus more conservative merging. Hence, the threshold *(th)* computed from the feature map generated through AttentionU-Net is more adaptive for merging over all the edge weights in the region adjacency graph (RAG) using equation 2.


$$\begin{array}{c} th\;=\;\mu \left(F_{I_{l}\;\in \;I}\right)|K| \end{array}$$ (2)

Where $\mu (F_{I_{l}\;\in \;I})$ represents the mean of the feature map generated by AttentionU-Net for the building ROI. The term $F_{I_{l}\;\in \;I}$ is calculated through equation 1. The |K| denotes the cardinality of the features vector, which means 1,..., *K,* and represents different buildings as ROI. Moreover, the MC based on the three (colour, texture, and shape) and fourth feature such as edge information, is incorporated into the merging process over the RAG generated by the SLIC OS. Based on the over-segmented image having adjacent regions such as $I_{r\;,}{\;I}_{j}\in I$, a RAG graph. The RAG graph consists of nodes in each over-segmented region and edges between adjacent over-segmented regions. Each node represents the calculated over-segmented region features vector for each over-segmented region. The edges in RAG represent the dissimilarity between adjacent regions $I_{r\;,}{\;I}_{j}\in I$. Hence, $I_{r\;,}{\;I}_{j}$ represents the two adjacent over-segmented regions in an over-segmented image. As presented in equation 3, $w_{\left(I_{r\;},\;I_{j}\right)\;\in \;I}$ will be the weight calculation formula of the edge connecting nodes on RAG representing regions $I_{r}$ and $I_{j}$, which corresponds to the dissimilarity between the over-segmented regions having features vector of the two adjacent regions, $I_{r}$ and $I_{j}$ in the over-segmented image. Moreover, $I_{j}$ represents the adjacent regions to $I_{r}$ in the over-segmented image.


$$\begin{array}{c} {E=w}_{\left(I_{r},\;I_{j}\right)\;\in \;I}=\sum \overset{O}{\underset{I_{r}=\text{1}}{\mathrm{\backslash nolimits}}}\sum \overset{b}{\underset{I_{j}=\text{1}}{\mathrm{\backslash nolimits}}}\left\|F_{I_{r}}-\;F_{I_{j}}\right\|\;\forall \;I_{r}\neq I_{j} \end{array}$$ (3)

Where $w_{\left(I_{r}\;,\;{\;j}_{r}\right)\;\in \;I}$ represents the edge weights between $I_{r}$ and $I_{j}$, which are the neighboring over-segmented regions. Moreover, *O* is the total number of over-segmented regions, and *b* is the total number of neighboring over-segmented regions for any given node $I_{r}$. While ∑ is used to calculate the total features values of over-segmented region, which are then subtracted from the adjacent over-segmented region using $\left\|F_{I_{r}}-\;F_{I_{j}}\right\|$. This results in a feature value that will be compared with the feature values of the building ROI generated feature map. Finally, in step sixth and seven, the MC represented as $M\left(I_{r}\;,\;I_{j}\right)\;$ will determine when to finally merge two nodes $I_{r}$ and $I_{j}$ based on the weight of the edge connecting them and the computed threshold as th through equation 2. Based on the above formulas, the following merging process has been performed, and hence MC can be represented as in equation 4.


$$\begin{array}{c} M_{\left(I_{r}\;,\;I_{j}\right)\;\in \;I}=\;\left\{ \begin{array}{c} \text{1}\;if\;w_{\left(I_{r},\;I_{j}\right)\;\in \;I}=min(w_{\left(I_{r},\;I_{j}\right)\;\in \;I})\;and\;w_{\left(I_{r},\;I_{j}\right)\;\in \;I}<\;th\\ \text{0}\;Otherwise\; \end{array}\right.\; \end{array}$$ (4)

**Fig 2:**
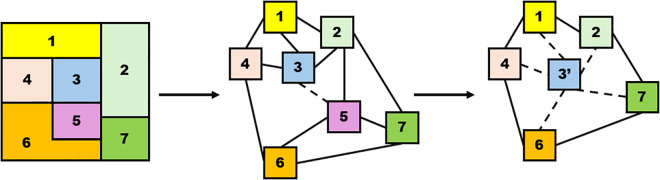
Construction of RAG and iterative region merging process in RAG. (a) initial OS, (b) regions merging threshold and MC checking, and (c) merged region and new region.

Where $min(w_{\left(I_{r}\;,\;I_{j}\right)\;\in \;I})\;$ is the minimum edge weight in the RAG and *th* represents the threshold. When the MC which is denoted with $M_{\left(I_{r}\;,\;{\;I}_{j}\right)\;\in \;I}$ is equals or less than the threshold, then regions $I_{r}$ and $I_{j}$ can be merged into a single region. If the difference of edge weights is below the threshold computed in equation 2 and satisfies the MC presented in equation 4, the two nodes merge to generate a new region. After merging nodes, the new region features are computed and assigned to new nodes in the RAG as demonstrated in Fig 2. Following each merge, the edges of the newly formed region are recalculated based on the four features of the merged regions. The region merging process continues until all the nodes in RAG are merged based on the above conditions. In the last step, initially colour features are grouped based on mean values similarity, followed by texture, which refines the grouping by considering surface patterns via Gabor filter matrices’ mean values. Afterwards, shape features further refine the region, having a number of pixels. Finally, edge features are used to precisely define boundaries, ensuring clear segmentation of the ROI. In brief, it means that the entire four features have been used to merge, but the three features (colour, texture, and shape) help with identifying and grouping the regions. Once this gets done, the fourth feature, which is edge information, will refine the segmentation by clearly marking the building region boundaries.

Fig 2 shows the concept of a RAG graph for region merging. Beginning with seven over-segmented regions as shown in Fig 2 (a), the nodes representing the over-segmented regions in Fig 2 (b), together with the edges connecting them, represent the relations between the regions. The MC formula of equation 4 assigns the weight of each edge as the dissimilarity of its two adjacent nodes. Afterwards, the threshold is then utilized to oversee the regions merging for over-segmented regions along with MC, which ensures that merging decisions are based on feature dissimilarity. Meanwhile, different buildings’ ROI will have different mean values, and for each building ROI, this will act as the threshold for iterative regions merging. For instance, the weight of the edge connecting nodes n3 and n5 in Fig 2 (b) is found to be the smallest, which means the nodes are highly similar. As a result, n3 and n5 will be merged into a new node, a new n3′, as shown in Fig 2 (c). The edge weights of node n3′ are subsequently changed. The process iteratively proceeds with the RAG graph updating until there are no regions left for merging. The following parameters, as demonstrated in Table 3, have been utilized for implementing the OS algorithm and DL architecture implementation for proposing the region merging approach in this study.

**Table 3:** Parameters for OS algorithm and DL architecture.

Algorithm/ Architecture	Parameters				
**SLIC k**	500	1000	1500	2000	3000
**AttentionU-Net**	epoch = 100	learning rate = 0.001	batch size = 6	drop out = 0.1	optimizer = Adam

## 4. Results and discussion

The performance evaluation results have been demonstrated in Tables 4-5 in terms of comparison between the integration of three (colour, texture, and shape) and four features (colour, texture, shape, and edge information) into MC for different SLIC algorithm k parameters. In addition, the F-measure, precision, recall, adapted rand index (ARI), adapted rand error (ARE), and variation of information (VOI), metrics have been employed. Furthermore, to find a better resultant feature integrated into MC, the visual comparison has been performed between three features as MC and Four features as MC. Finally, the proposed region merging approach and previous work are compared by utilizing metrics which are $G_{s}$, F-measure, precision, and recall.

**Table 4:** Results of three features as MC for parameter k in SLIC value 500, 1000, 1500, 2000, 3000, respectively.

Input image No.	OS value of k for SLIC	No. of Regions Generated	Evaluation Metrics					
			F- measure	precision	recall	ARI	ARE	VOI
**101**	500	250	0.39	0.82	0.3	0.65	0.6	1.42
	1000	612	0.62	0.80	0.5	0.76	0.37	1.11
	1500	940	0.68	0.83	0.5	0.79	0.31	0.98
	2000	1421	0.77	0.85	0.7	0.83	0.22	0.88
	**3000**	**2165**	**0.84**	**0.89**	**0.8**	**0.86**	**0.15**	**0.75**
**829**	500	234	0.57	0.43	0.8	0.7	0.42	0.29
	1000	554	0.88	0.79	0.9	0.85	0.11	0.20
	1500	902	0.94	0.89	0.9	0.91	0.05	0.12
	2000	1324	0.95	0.92	0.9	0.93	0.04	0.09
	**3000**	**2068**	**0.98**	**0.9**	**0.9**	**0.95**	**0.01**	**0.07**
**1027**	500	303	0.89	0.84	0.9	0.88	0.10	0.30
	**1000**	**661**	**0.95**	**0.92**	0.9	0.91	**0.04**	0.24
	1500	1027	0.95	0.91	**0.9**	0.92	0.04	0.21
	2000	1538	0.93	0.88	0.9	0.92	0.06	0.22
	3000	2324	0.94	0.89	0.9	**0.93**	0.05	**0.20**

**Table 5:** Results of four features as MC for parameter k in SLIC value 500, 1000, 1500, 2000, 3000, respectively.

Input image No.	OS value of k for SLIC	No. of Regions Generated	Evaluation Metrics					
			F-measure	precision	recall	ARI	ARE	VOI
**101**	500	250	0.87	0.89	0.8	0.72	0.14	0.62
	1000	612	0.87	0.89	0.8	0.77	0.12	0.57
	1500	940	0.88	0.90	0.8	0.80	0.10	0.46
	2000	1421	0.89	0.90	0.8	0.86	0.09	0.39
	**3000**	**2165**	**0.90**	**0.91**	**0.8**	**0.91**	**0.08**	**0.35**
**829**	500	234	0.79	0.75	0.8	0.81	0.09	0.08
	1000	554	0.93	0.90	0.9	0.88	0.04	0.06
	1500	902	0.95	0.92	0.9	0.92	0.03	0.05
	2000	1324	0.97	0.96	0.9	0.95	0.01	0.03
	**3000**	**2068**	**0.99**	**0.99**	**0.9**	**0.97**	**0.01**	**0.03**
**1027**	500	303	0.92	0.89	0.9	0.95	0.08	0.17
	1000	661	0.95	0.93	0.9	0.95	0.09	0.14
	1500	1027	0.98	0.96	0.9	0.97	**0.03**	**0.09**
	2000	1538	0.96	0.94	0.9	0.97	0.04	0.10
	**3000**	**2324**	**0.98**	**0.97**	**0.9**	**0.98**	0.03	0.11

Subsections 4.1 and 4.2 below have demonstrated the results for the specified SLIC algorithm k parameter for initial OS generation by incorporating three and four features into MC, respectively.

### 4.1. Results for three features with specified k parameter value in SLIC

The performance evaluation results of incorporating three features into MC for different k parameter values in SLIC are presented in Table 4. These values for parameter k are 500, 1000, 1500, 2000, and 3000, respectively. As presented in Table 4, the three features incorporated in MC, image ID 101, have achieved an F-Measure of 0.84 through the SLIC algorithm with parameter k = 3000 by generating 2165 over-segmented regions. The same image with the k *=* 3000 parameter of SLIC achieved values of 0.86 for ARI, 0.15 for ARE, and the VOI value reached 0.75, respectively.

The image ID 829 has demonstrated the F-measure value of 0.98 via the SLIC algorithm k parameter of value 3000, by generating 2068 over-segmented regions. While the same image with achieved values of 0.95 for ARI, ARE is 0.01, and VOI is 0.07, with the SLIC k *=* 3000, respectively. The image ID 1027 has shown the value 0.95 for the F-measure metric by setting SLIC k = 1000, which generated over-segmented regions of 661. Along with this, the same image with achieved values of ARE is 0.04. However, for ARI and VOI, the values are 0.93 and 0.20, with the SLIC k *=* 3000, respectively.

### 4.2. Results for four features with specified k parameter value in SLIC

As demonstrated in Table 5, for utilizing the four features, which are colour, texture, shape, and edge information incorporated into MC from the AttentionU-Net generated feature map. The results of different initial OS have been demonstrated by employing MC during the regions merging. The image ID 101 has achieved an F-measure of 0.9040 through SLIC with parameter k = 3000 by generating 2165 over-segmented regions. For the same image, having parameter k *=* 3000 of SLIC achieved values of 0.91 for ARI, 0.08 for ARE, and a VOI value is reached to 0.35, respectively.

The image ID 829 has demonstrated the F-measure value of 0.99 for the SLIC k parameter of value 3000 by generating 2068 over-segmented regions. Additionally, the same image has achieved values of 0.97 for ARI, ARE is 0.01, and VOI is 0.03, with the SLIC k *=* 3000, respectively. The image ID 1027 has shown the value 0.98 for the F-measure metric by setting SLIC k = 3000 by generating a number of 2324 over-segmented regions. Additionally, the same image achieved ARI values of 0.98. The metric ARE demonstrated 0.03, and VOI shows 0.09 with the SLIC k *=* 1500, respectively.

**Fig 3:**
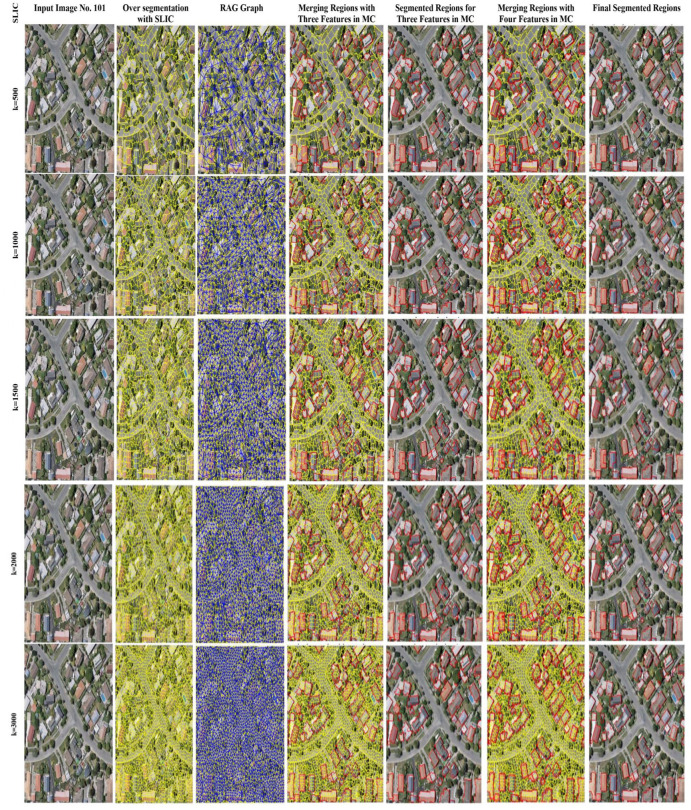
SLIC Parameter value (k). Column containing input image 101 is base imagery from the WHU Buildings Dataset [12], visualizations, and segmentation outputs.

**Fig 4:**
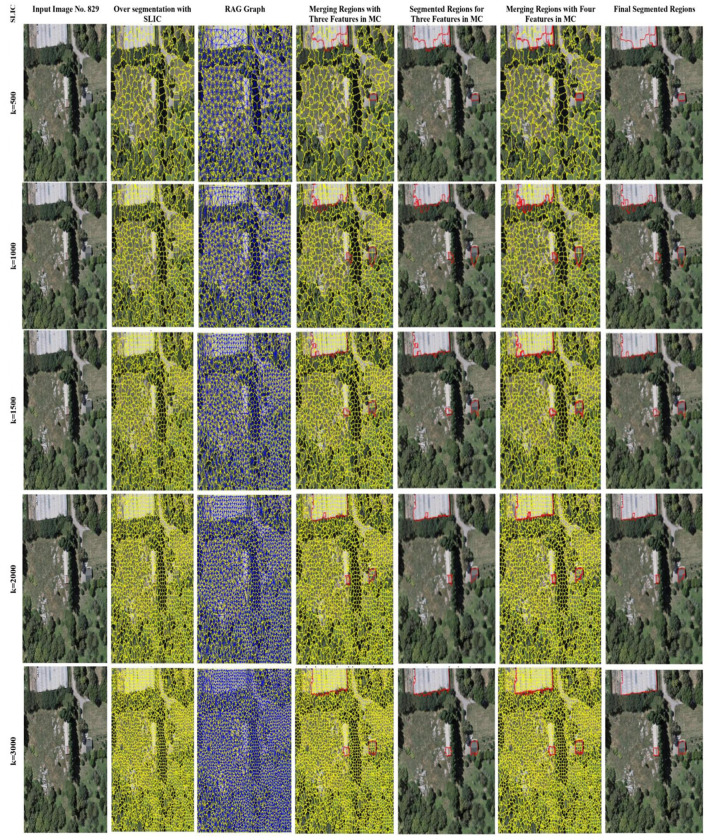
OS via SLIC having parameter k value (k = 500, 1000, 1500, 2000, and 3000). Column containing input image 829 is base imagery from the WHU Buildings Dataset [12], visualizations, and segmentation outputs.

**Fig 5:**
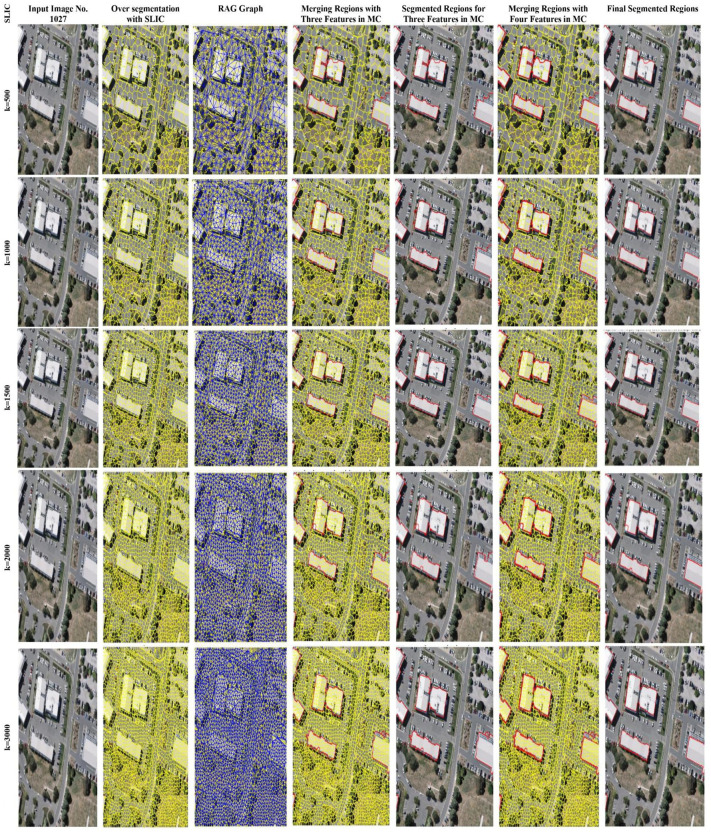
SLIC Parameter value (k). **Column containing input image 1027 is base imagery from the WHU Buildings Dataset [** 12 **], visualizations, and segmentation outputs.**

Furthermore, from Figs 3-5, for image IDs 101, 829, and 1027, the initial OS, subsequent RAG generation, and three and four features as MC for region merging have been demonstrated, respectively. The visual results indicate that the SLIC algorithm, having parameter k values of 500, 1000, 1500, and 2000, has missed some building regions either completely or partially for three features as MC as well as for four features as MC, respectively. The k values 1500 and 2000 have segmented the buildings’ ROI, but due to some inaccurate boundaries identified for regions as compared to the k parameter value 3000. Although, k parameter value 3000 of the SLIC algorithm also contains some missed or irregular boundaries, even though the edge information is present in the AttentionU-Net-generated feature map to provide accurate boundaries. The edge features implementation is based on the satisfaction of three features, MC, and once the three features incorporated provides an initial merging range, then the edge information assists in uniforming those boundaries which have high similarity with the over-segmented region. It means that even though the edge features from the AttentionU-Net generated feature map and the over-segmented region are included in the feature vector, they will not merge outside the three-feature range. This is due to the strength of the information between the edges. As can be seen in Figs 3-5 of the SLIC algorithm, with parameter k = 500, the algorithm has missed two buildings in the top-centre of the image, the centre building, and the bottom-left, respectively, for image IDs 101, 829, and 1027. Although the buildings in the top-centre of the image, the centre building, and the bottom-left are present in the AttentionU-Net generated feature map as well as the edge features intensity map, respectively.

**Fig 6:**
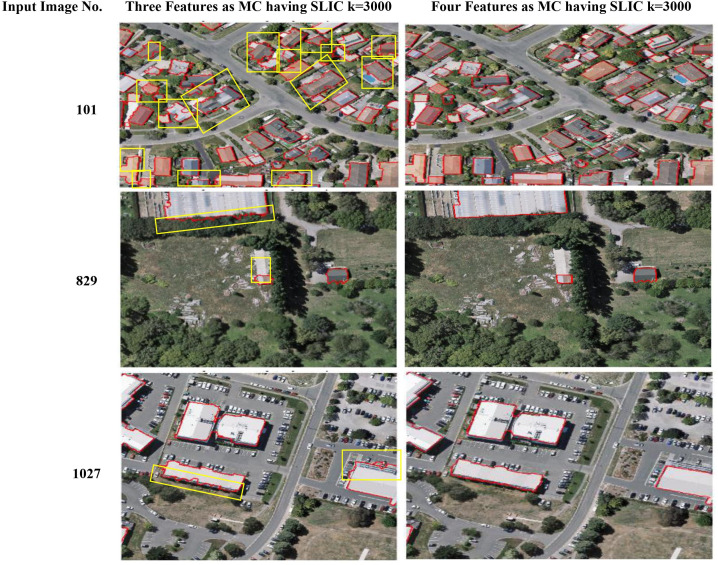
Comparison of segmentation results by incorporating three and four features into MC, respectively. Rows containing input image 101, 829, 1027 are visualizations and segmentation outputs.

As mentioned in the literature, the region growing algorithm starts from within the objects and finishes by identifying the boundaries. Hence, this proposed region merging process has similarity to the region growing algorithm. In this proposed region merging approach, if initially the edge features are integrated, and then the other three features mentioned above are utilized to perform merging. Then the chances are there that the building’s ROI feature map generated by AttentionU-Net has edges that consist of extra pixels; such edges would influence the region merging, and resultantly, the building segmentation would be inaccurate. Based on the comparisons demonstrated in Tables 4-5, and the F-measure values as well as the visual results from Figs 4-6, this explores the generation of initial OS. While different k parameter values of the SLIC algorithm are applied, the results are compared using both three and four-feature-based MC. The findings demonstrated that the SLIC algorithm provides better results by using parameter k = 3000.

The process of incorporating edge features along with three features will work as initially the colour, texture, and shape features criterion will be satisfied and based on which the edge features will assist in generating the boundaries within the generated or satisfied colour, texture, and shape region in terms of generating uniform edges. It means that once the three features satisfy the process of merging regions, the edge features map would fulfil the region boundaries within the merged region and would produce the uniform boundaries. This way, the resultant image would have straight boundaries by incorporating four features into MC as compared to three features. Meanwhile, to be more specific, the colour, texture, and shape features will select the relevant features of the object and later will be uniformed with edge information. During the region merging process, colour, texture, shape, and edge features collectively enhance segmentation.

### 4.3. Result analysis on the specified k parameter value in SLIC by incorporation of three and four features as MC

From the results presented in Table 4-5, as well as the difference in F-measure between Tables 4-5 is also notable. As from the results in Table 5, average F-measure values for all the images have achieved a higher value as compared to Table 4. One of the reasons for achieving higher values in Table 5 is the incorporation of the features into MC. It means that the four features of integration have significantly impacted the final segmentation outcomes. It is because the more features are utilized, the better the segmentation results will be. Tables 4-5 present three and four features results as MC, respectively. The metrics used for comparison between three and four features, as MC, are F-measure, precision, recall, ARI, ARE, and VOI. The results of both Tables 4-5 prove that SLIC with parameter k = 3000 outperform among k values of 500, 1000, 1500, and 2000. Furthermore, from Fig 3-5, the segmentation results of parameter k *with* different values in the SLIC algorithm have been presented visually. Moreover, Figs 3-5 demonstrate the visual comparisons, and it has been observed that the initial OS has an influence on segmentation output. The results indicate that the higher the OS, the better the final segmentation.

### 4.4. Comparison of three and four features as MC

The results in Table 6 demonstrate the comparison between three features and four features employed in MC for different values of parameter k in SLIC. While the SLIC algorithm with parameters k = 500, 1000, 1500, 2000, and 3000 is utilized to generate the initial OS. The best results achieved with k = 3000 as average results for F-measure on the WHU Buildings dataset [38] for three features achieved 0.88, ARI as 0.89, ARE as 0.11, and VOI as 0.50, respectively. The four features acting as MC produce the average results on the WHU Buildings dataset for F-measure of 0.91, ARI of 0.76, ARE of 0.07, and VOI of 0.39, respectively.

**Table 6:** Average results for three and four features incorporated as MC for SLIC parameter k value 500, 1000, 1500, 2000, 3000, respectively, on 1550 images of the WHU Buildings Dataset.

Features into MC	OS Value of parameter k for SLIC Algorithm	Evaluation Metrics					
		F-measure	precision	recall	ARI	ARE	VOI
**3 features**	500	0.63	0.69	0.58	0.57	0.20	0.64
	1000	0.66	0.71	0.61	0.69	0.18	0.57
	1500	0.71	0.76	0.67	0.79	0.17	0.55
	2000	0.75	0.79	0.72	0.80	0.16	0.54
	**3000**	0.88	0.88	0.88	**0.89**	0.11	0.50
**4 features**	500	0.89	0.90	0.88	0.64	0.15	0.49
	1000	0.90	0.91	0.89	0.69	0.13	0.30
	1500	0.90	0.91	0.89	0.73	0.12	0.15
	2000	0.90	0.91	0.89	0.76	0.10	0.06
	**3000**	**0.91**	**0.92**	**0.90**	0.76	**0.07**	**0.39**

### 4.5. Analysis on three and four features incorporation into MC

It has been observed in Tables 4-5 that, the SLIC algorithm parameter k = 3000 shows better results in terms of performance evaluation, respectively. While, in Table 6, SLIC parameter k = 3000 also achieved best results on WHU Buildings Dataset [38]. The comparison between the integration of three and four features into the MC demonstrates that incorporating four features, which are colour, texture, shape, and edge, has achieved better results than using only three features. Moreover, the results show that four features, which include colour, texture, shape, and edge feature, provide better segmentation results as compared to three features in MC. It means that when the initial OS is excessive, then the features employed in MC are most correlated to regions in the over-segmented region. In this way, it becomes easy for the MC to be satisfied and merge regions, hence providing better segmentation results. Fig 6 further confirms this from its visual results. The buildings which are extracted with three features have displayed some irregular boundaries, while the inclusion of the edge feature has produced more uniform and accurate building boundaries. The improvement is caused by the edge feature from the AttentionU-Net building feature map, which helps in identifying the boundaries close to over-segmented regions in the image, thus refining the results once the colour, texture, and shape features have established the initial merging range. Mentioning that the edge feature does not extend beyond the range that is defined by the three core features, thus ensuring precision in region merging. Hence, this experimental analysis has shown that integration of four features enhances the segmentation quality. Thus, the research has selected SLIC parameter k = 3000 with four features in MC for comparison with the previous region merging approaches.

### 4.6. Comparison with state-of-the-art segmentation algorithms

**Fig 7:**
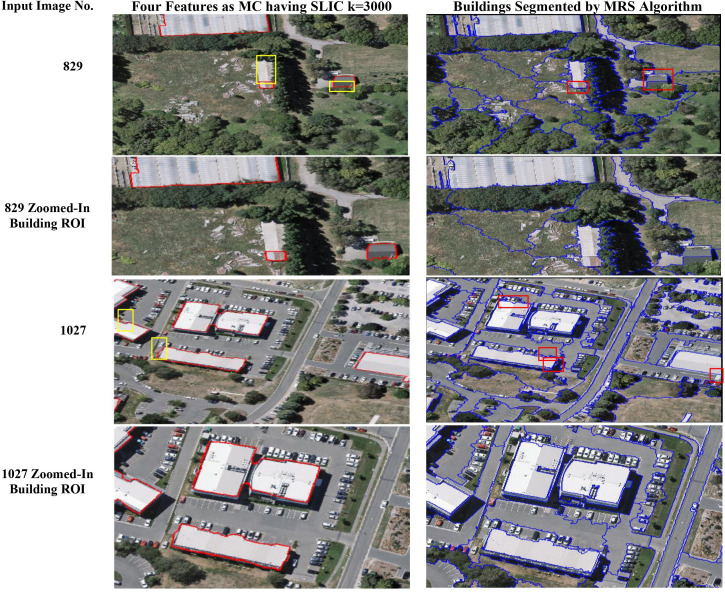
Comparison of final segmentation results with the utilization of four features into MC with the MRS algorithm [47] on the WHU Buildings dataset [38]. Rows containing input image 829 and 1027 are visualizations and segmentation outputs.

Table 7 shows the comparison of the proposed region merging approach with the previous methods in the literature [47], on the WHU Buildings RS images dataset (1550 images) [46]. The widely used multiresolution segmentation (MRS) algorithm, a bottom-up region merging approach [47], implemented in eCognition software, is based on three parameters, which are scale, shape, and compactness. For this research, the MRS algorithm has been tested with a scale parameter of 130 and shape and compactness set to 0.5, following the [46] paper. Even though the previous research [46] has achieved an average F-measure of 0.63, a precision value of 0.82, and a recall of 0.52, the proposed approach has significantly outperformed it with an average F-measure value of 0.91, a precision of 0.92, and a recall value of 0.90. Similarly, the proposed approach has achieved a higher $G_{s}$ value (0.92) as compared to MRS (0.83), therefore, demonstrating superior segmentation results on the WHU dataset. Fig 7 shows the visual comparison of the proposed region merging approach with the MRS algorithm [47] on the WHU Buildings dataset. For image ID 829, the proposed approach has produced more accurate building segmentation results, while the MRS has either missed parts of the ROI or segmented extra regions, as highlighted in the yellow boxes in the images. Similarly, in image ID 1027, the proposed approach has shown a slight US, whereas the MRS here has resulted in OS. These differences arise because the paper [46] is focused on building ROIs, while the MRS [47] segments other objects in images as well, making the direct comparison difficult. Thus, the $G_{s}$ metric was used for the evaluation of building delineation between the two approaches.

**Table 7:** Comparison of final segmentation results of the proposed approach with previous state-of-the-art research work. Results of three features as MC for parameter k in SLIC value 500, 1000, 1500, 2000, 3000, respectively.

Ref	Initial Segmentation Algorithm	Metrics Used	Avg F-measure	Avg precision	Avg recall	Average $G_{s}$
[48]	High-Quality Segment Anything Model (HQ-SAM) in a zero-shot model	F-Measure, Precision, and Recall.	0.90	0.90	0.89	---
[46]	Watershed transformation		0.63	0.82	0.52	
[47]	Region Growing	$G_{s}$ (Goodness for segmentation)	---	---	---	0.83
**Proposed approach/ Four Features as MC**	SLIC algorithm parameter k = 3000 value	F-Measure, precision, and recall. -- $G_{s}$ (Goodness for segmentation)	**0.91**	**0.92**	**0.90**	**0.92**

### 4.7. Analysis of the proposed region merging approach in comparison to previous research works

Table 7 results demonstrate that the proposed region merging approach has achieved higher F-measure, precision, and recall values as compared to previous work [46]. This improvement is attributed mainly to the preprocessing of the WHU Buildings dataset [38], which was not performed in the following research [46], as well as the utilization of four features, which are colour, texture, shape, and edge, in MC instead of only the spectral angle. Likewise, the MRS algorithm [47] has achieved a lower $G_{s}$ as compared to the proposed methodology since it relies on image colour, texture, and smoothness. The inclusion of edge features in this research has significantly enhanced segmentation accuracy. Fig 7 provides a visual comparison for further validation of these results; both approaches segment buildings effectively, but the proposed methodology reduces under-segmentation (US) and also avoids over-segmentation, which is observed in MRS. Limitations faced in the MRS algorithm include difficulty in threshold selection and in distinguishing between similar colours, such as roofs and roads, which often leads to segmentation errors. In contrast to this, the proposed approach in this research work benefits from AttentionU-Net-based feature maps for threshold optimization and MC derivation. This strategy eliminates the need for human intervention and thus enables more efficient and accurate region merging. Consequently, for building delineation in RS images, the proposed approach provides superior segmentation results.

## 5. Conclusion

In this study and experimentation, a region merging approach is proposed in order to delineate the buildings regions as ROI in the WHU Buildings RS images dataset. In the proposed approach, AttentionU-Net, a CNN-based DL architecture, is utilized for generating the feature map of the buildings as ROI in the RS images. From this obtained feature map, prominent features of the ROI, including the colour, texture, shape, and edge features, were extracted. These features are then used for threshold optimization and deriving the MC using over-segmented regions generated from the SLIC algorithm for merging without any intervention by humans.

The proposed approach for region merging has achieved a higher average F-measure value of 0.91 in comparison to a similar existing literature work that required human intervention for merging buildings regions in the WHU Buildings RS images dataset [46], which has achieved an average F-measure value of 0.63. Furthermore, in comparison to another similar work, which is the MRS algorithm [47], the proposed approach achieved an average $G_{s}$ of 0.92. While the MRS algorithm scored an average $G_{s}$ of 0.83 for delineating building regions in the same dataset. The higher results of the proposed region merging approach are mainly because of the feature map generated by the AttentionU-Net. This feature map accurately and precisely defines buildings as ROI. Later on, allowing the subsequent feature extraction process to select the prominent features for deriving the MC to perform the merging without any human intervention. The proposed region merging approach proves the best results for building ROI delineation as compared to previous research works [45,46].

In RS image segmentation, the outputs of DL as well as conventional algorithms have great effects on the performance outcomes based on the data properties and image pre-processing. Generally, the DL architectures perform better on similar datasets rather than different ones, it is because of the domain difference. In domain difference, the pre-trained DL architecture, when applied to different data or images, faces problems due to a sudden change of properties or characteristics in data [49]. Thus, in our proposed approach, this is one of the drawbacks that the AttentionU-Net trained on the WHU Buildings RS images dataset faces difficulty of generalization on other datasets and resulting in the feature extraction part being incomplete. As feature extraction is one of the core dependencies of our proposed region merging approach.

### 5.1. Limitations and future work

In the proposed region merging approach, there are still some instances where the AttentionU-Net CNN-based DL architecture used has failed to generate a feature map for small buildings regions. Resultantly, these smaller regions will not be delineated by this proposed approach. This is an indication that the feature map, which is produced by the AttentionU-Net model, will have a significant impact on the process of feature extraction and the MC derivation for merging the buildings regions. Therefore, the proposed region merging approach may not be ideal for real-time buildings segmentation in remote sensing image analysis. The limitation is due to the dependence on the AttentionU-Net, which requires extensive training that results in delays in the iterative merging process.

For future work, in order to address the challenges for training the AttentionU-Net, a transfer learning technique can be developed for the generation of a feature map with a reduced number of training images to minimize delays in the merging process. In addition, the AttentionU-Net can also be integrated with some additional attention mechanisms, like residual networks such as ResNet, for enhancing its capabilities in the generation of a feature map for small object regions, such as ROI in RS images.
